# Teriparatide treatment in severe osteoporosis – a controlled 10-year follow-up study

**DOI:** 10.1186/s12891-022-05987-2

**Published:** 2022-11-24

**Authors:** Georgios Kontogeorgos, Emily Krantz, Penelope Trimpou, Christine M. Laine, Kerstin Landin-Wilhelmsen

**Affiliations:** 1 Department of Medicine, Section for Geriatrics and Emergency Medicine, Sahlgrenska University Hospital/Östra, Gothenburg, SE-41650 Gothenburg, Sweden; 2 Institute of Medicine, Department of Internal Medicine and Clinical Nutrition, Sahlgrenska Academy, University of Gothenburg, Gothenburg, Sweden; 3 Department of Respiratory Medicine and Allergology, Sahlgrenska University Hospital, Gothenburg, Sweden; 4 Section for Endocrinology, Department of Medicine, Sahlgrenska University Hospital, Gothenburg, Sweden; 5 Endocrine Out-Patient Clinic, Hospital of Halland, Kungsbacka, Sweden

**Keywords:** Teriparatide, Osteoporosis, Fractures, Quality of life

## Abstract

**Background:**

Teriparatide was the first anabolic agent recommended for the treatment of osteoporosis. Long-term real-world, controlled studies are not available. The purpose was to evaluate the long-term effects of treatment with teriparatide on fractures and Health Related Quality of Life in subjects with established osteoporosis in comparison with placebo treated patients with osteoporosis and the general population.

**Methods:**

A 10-year follow-up was performed after a prospective, open-labelled study with teriparatide 20 μg given subcutaneously daily for a mean of 18 months (range 14–24 months) in 40 women, mean age 69 years, with osteoporosis and vertebral compression. Placebo treated women, *n* = 25, mean age 60 years, from a randomized, double-blind, placebo-controlled growth hormone trial with daily subcutaneous injections for 18 months, with osteoporosis were used as controls. Dual energy x-ray absorptiometry and questionnaires were performed at start, after 18 months, after 36 months and after 10 years. Women, *n* = 233, of similar age from a random population sample, also served as controls and were followed in parallel. All fractures were X-ray verified.

**Results:**

Fractures decreased from 100 to 35% in the teriparatide treated patients (*p* < 0.0001) to similar levels as in the population sample, 25 to 28% at start and after 10 years, respectively. Bone mineral density increased on teriparatide but returned to levels at treatment start after 10 years. Health Related Quality of Life was lower in the teriparatide group than in the population (*p* < 0.001) before and, after treatment and at 10 years.

**Conclusions:**

Anabolic hormonal treatment with teriparatide reduced fracture prevalence to similar levels as in the general population at 10 years’ follow-up. Health Related Quality of Life was low in osteoporosis and unaffected by bone specific treatment.

## Background

Fractures are an increasing problem and cause of morbidity and mortality in the population [1]. Despite advances in diagnosis and treatment, osteoporosis is still a hidden disease and a vertebral or peripheral fracture is often the first sign of the presence of fragile bone [2]. Few agents are available that stimulate osteoblasts activity to increase bone mass. Teriparatide was approved by the European Medicines Agency in 2003 as the first anabolic agent for established osteoporosis. Teriparatide has been shown to reduce the risk for new vertebral compressions by up to 65% and increase bone mineral density (BMD) by 9% in the lumbar spine [3]. A clinical trial studying the effect of growth hormone treatment in women with postmenopausal osteoporosis was also published in 2003 that showed improved BMD with similar magnitude as the in teriparatide study [4]. Both are anabolic agents and are administrated subcutaneously once a day [3, 4].

The use of an antiresorptive treatment is recommended after completing teriparatide treatment in order to preserve the newly formed bone [5]. Safety considerations have limited the use of teriparatide because of reported cases of osteosarcoma in rodents treated with very high doses of teriparatide [6, 7]. Osteosarcoma has not been identified in any humans treated with teriparatide so far, according to an ongoing prospective register study [8]. Different doses were tested in studies but the benefits of the treatment with a dose exceeding 20 μg daily were not significant enough to justify the higher frequency of side effects [3].

Health Related Quality of Life (HRQoL) is impaired after a fragility fracture [9, 10]. In patients treated with teriparatide, HRQoL was improved in a shorter previous uncontrolled follow up study [11].

In the present study, a 10-year follow up is presented for patients treated with teriparatide for a mean of 18 months (range 14–24 months). They were compared with patients treated with placebo injections during 18 months in a previous randomized, double-blind, placebo-controlled study [4] and followed for 10 years. Fractures and HRQoL were studied at baseline and 10 years later. A population sample from the same city was also followed in parallel and used as a reference group.

## Methods

### Design

A prospective, open-label treatment study for osteoporosis with teriparatide was started in 2004. The results from the teriparatide study were compared with placebo treated patients from a randomized, double-blind, placebo-controlled prospective study with growth hormone, administrated to postmenopausal women with osteoporosis. During the follow-up period women from a randomly selected population, the World Health Organization MONItoring of CArdiovascular (WHO MONICA) project in Gothenburg, served as population-based controls followed in parallel.

### Patients with osteoporosis treated with teriparatide

**Fig. 1:**
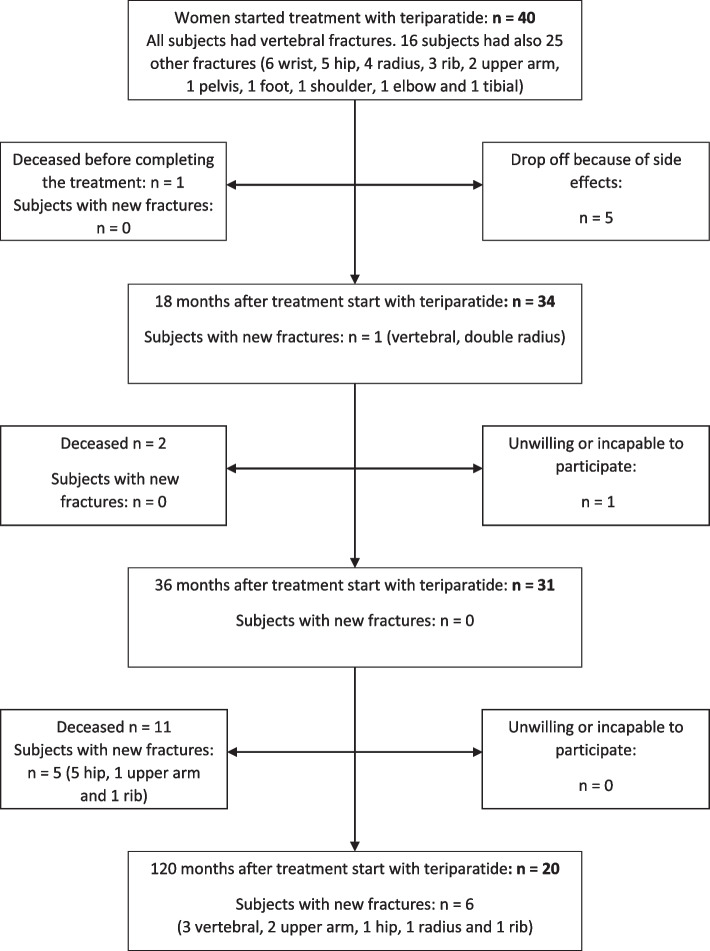
Flow chart showing the 10 years follow-up of women with osteoporosis treated with teriparatide for a mean of 18 months, range 14–24 months. All women had at least one vertebral compression at baseline. The number of women who suffered a fracture and the type of fracture, at baseline and during the entire treatment time and follow-up are depicted in each box

Women, *n* = 40, mean age 69 + 11 years, with established osteoporosis and at least one vertebral compression fracture were included consecutively from 2004 until 2013 after referrals from primary care physicians, orthopedists or other bone specialists in western and southern region of Sweden. Before teriparatide, 57% had treatment with bisphosphonate, 20% had estrogen hormone replacement treatment (HRT), and 25% had corticosteroid treatment of which 8% had hydrocortisone substitution due to pituitary insufficiency. Subjects starting treatment with teriparatide were included in the SweFOS study, later renamed to ScanFOS and ExFOS, respectively [12]. After clinical evaluation, and if there were no contraindications for the treatment, patients were given information, signed informed consent, were instructed regarding the injection technique by a research nurse and started the subcutaneous treatment with teriparatide 20 μg daily for up to 2 years. The recommended treatment duration changed from 18 to 24 months in 2016 [13]. Calcium 1000 mg and cholecalciferol 800 IU were given daily. The same endocrinologist, at the Sahlgrenska University Hospital Endocrine Outpatient Clinic had the responsibility for treatment with teriparatide and patients’ follow-up with annual examinations during 10 years. When needed, patients received bisphosphonates after the teriparatide treatment. Flow chart is shown in Fig. 1.

### Controls with osteoporosis treated with placebo injections

For comparison of the treatment effect on BMD, women with postmenopausal osteoporosis, treated with placebo *n* = 25 for 18 months, in the frame of a previous study with growth hormone [4], were used and were followed for 10 years. Detailed description of the previous study is published elsewhere [4]. Briefly, patients participated in a randomized, placebo-controlled, double-blind study, with growth hormone subcutaneously 1.0 IU/day *n* = 28, 2.5 IU/day *n* = 27 and similar volumes of placebo *n* = 25. All women received 750 mg calcium and 400 IU cholecalciferol daily [4]. HRT was an inclusion criterion and recommended for osteoporosis according to the guidelines at that time [14]. All had Dual energy X-ray Absorptiometry (DXA)-verified osteoporosis and in the placebo group 14% had a previous fracture according to the medical history or vertebral X-ray [4]. All participants signed an informed consent and were followed for 10 years by the same investigator, with annual examinations. None dropped out. When needed, patients received bisphosphonates.

### Controls from the general population

In 1995, 2400 men and women aged 25–64 years were randomly selected from the city census of Gothenburg to participate to an international study, the WHO MONICA study, which was designed to compare risk factors and the incidence of cardiovascular diseases in 38 different populations around the world [15]. In Gothenburg, the principal investigator added an endocrine examination with hormonal biochemistry and bone measurements in every fourth man 25–64 years, and every fourth woman aged 25–44 and all women aged 45–64, *n* = 662, with the objective to serve as a control group for endocrine diseases and fractures, predominantly female disorders. Of the 662 subjects included in the additional endocrine and bone examinations 608 were alive in 2007 and 414 participated (68%) in a re-examination (77% women) [16]. Of the 414 participants, a random subgroup of women (*n* = 233) was matched for age with patients treated with teriparatide. Of those, 177 women (75%) were alive in 2020. Radiologically verified fractures, based on their medical records, were compared with the fractures in patients treated with teriparatide during a similar period of time. HRT was used by 22%, bone specific agents by 4.5% and calcium/vitamin D by 14%.

### Anthropometry

Body weight was measured to the nearest 0.1 kg with light clothing. Body height was measured to the nearest 1.0 cm without shoes. Body mass index (BMI) was calculated as body weight in kilograms (kg) divided by height squared in meters (m) and presented as kg/m^2^. Waist circumference was measured by the same observer in each study with a soft tape between the lowest rib margin and the iliac crest in the standing position. The hip circumference was also measured with a soft tape over the widest part of the gluteal region and the waist-to-hip ratio was calculated.

### Bone measurement methods

DXA was performed at the same laboratory for all patients at start and follow up with LUNAR DPX-L; Lunar Radiation Inc., Madison, WI, USA.

The DXA scan was changed to GE Medical Systems, Lunar Corp., Madison, WI, USA, with the use of iDXA hardware, in 2015 for the 10-year follow up in the teriparatide study.

### Fractures

Radiologically verified fractures were monitored at start and every annual visit during 10 years among the patients with osteoporosis. Fractures, X-ray verified in the medical record, were also asked for at start and after 10 years in the WHO MONICA population sample. All participants were followed up at the Sahlgrenska University Hospital Endocrine Outpatient Clinic.

### Biochemistry

Blood samples were drawn in the morning at fasting state from an antecubital vein. Blood samples were analyzed at the accredited laboratory of Sahlgrenska University Hospital. Total serum calcium (S-Ca total), serum ionized calcium (S-Ca ion), serum parathyroid hormone (S-PTH) and serum osteocalcin (S-Osteocalcin) were used to monitor patients. S-Ca total was analyzed with photometry, S-Ca ion with ion-selective electrodes, S-PTH with IRMA (Roche Cobas, Rotkreutz, Switzerland). Reference levels were for S-Ca total, 2.15–2.50 mmol/L, S-Ca ion, 1.15–1.31 mmol/L, S-PTH, 1.6–6.9 pmol/L and S-Osteocalcin for menopausal women 5.4–59 μg/L and for men 4.6–65 μg/L.

### HRQoL questionnaire

EuroQol-5 Dimensions Visual Analogue Scale questionnaire (EQ5D-VAS), was used to estimate the HRQoL [17]. EQ5D-VAS consists of two sections. In the first section patients evaluate their health status in five domains (mobility, self-care, usual activities, pain/discomfort, and anxiety/depression) in a three-point scale which reflects the grade of the severity of their status (no problems, some problems, and severe problems). In the second section patients rate their health status in a scale from 0 to 100 mm, with 0 mm representing the worst imaginable state of health, and 100 mm the best state of health. The questionnaires were completed by patients with teriparatide treatment at the baseline, at 18, 36, and 120 months and at baseline in the WHO-MONICA population-based control group. A score of 80 mm or above was used as cut-off for a good self-rated health as previously reported [18].

### Statistical analyses

Means and standard deviations (SD) were calculated using IBM SPSS statistics version 26 (IBM, NY, USA). For comparison between groups, two tailed t-test and the Fisher’s exact test was used for dichotomous variables. Paired t-test was used for comparison between same patients at start and at the end of the follow up period. The Mann-Whitney test was used for non-parametric variables. Chi-square test with Yates correction was used for comparison between different groups. A *p*-value of < 0.05 was considered statistically significant.

## Results

Of the 40 women, mean age at start 69 + 11 years, 20 women, completed the 10-year follow-up in the teriparatide study and had a mean age at start 64 + 12 years (Fig. 1). Fourteen women (35%) died during follow-up. Baseline characteristics for the entire group (*n* = 40) at start and for the 20 patients who were examined 10 years later are described in Table 1. Body height and number of fractures decreased (Table 1).

**Table 1:** Anthropometric data, biochemistry, fractures and agents that affect bone metabolism for patients treated with teriparatide at the start of the treatment, for all women (*n* = 40) and for all women with 10 years follow-up (*n* = 20). The *p*-value shows comparison between study start and 10 years’ follow-up in the 20 women who fulfilled the entire teriparatide study

Anthropometric and treatment data Mean (Standard Deviation)	Teriparatide at treatment start ***n*** = 40	Teriparatide at treatment start ***n*** = 20	Teriparatide 10 years after treatment start ***n*** = 20	***p***-value 10 years versus start ***n*** = 20
Age (years)	69.5 (10.9)	64.2 (12.3)	74.7 (12.5)	**< 0.001**
Height (m)	1.59 (0.86)	1.60 (0.91)	1.58 (0.11)	**0.003**
Body weight (kg)	60.3 (10.2)	61.6 (11.7)	60.8 (16.2)	0.62
BMI (kg/m^2^)	24.0 (3.7)	23.7 (4.1)	24.3 (5.0)	0.25
Waist (cm)	86.8 (10.5)	86.7 (9.3)	90.6 (14.7)	0.14
Hip (cm)	101.5 (7.6)	102.2 (9.5)	100.6 (13.5)	0.55
Waist to hip ratio	0.86 (0.09)	0.87 (0.07)	0.91 (0.10)	0.51
BMD femoral neck (g/cm^2^)	0.68 (0.15)	0.68 (0.17)	0.69 (0.20)	0.97
BMD lumbar spine(g/cm^2^)	0.89 (0.20)	0.88 (0.22)	0.93 (0.25)	0.62
T-score femoral neck	−2.60 (1.16)	−2.58 (1.30)	−2.59 (1.63)	0.62
T-score lumbar spine	−2.61 (1.69)	−2.60 (1.82)	− 2.25 (2.09)	0.39
S-Osteocalcin (μg/L)	12.8 (6.3)	14.9 (6.8)	21.6 (26.0)	0.55
S-Ca total (mmol/L)	2.32 (0.09)	2.29 (0.06)	2.37 (0.11)	**0.02**
S-Ca ion (mmol/L)	1.23 (0.07)	1.20 (0.08)	1.25 (0.04)	0.26
S-PTH (pmol/L)	4.3 (1.0)	4.1 (0.7)	3.8 (1.4)	0.13
Subjects with fractures, n (%)	40 (100%)	20 (100%)	7 (35.0%)	**< 0.001**
Cortisone treatment, n (%)	10 (25.0%)	4 (20.0%)	4 (20.0%)	1.00
Estrogen replacement, n (%)	8 (20.0%)	4 (20.0%)	2 (10.0%)	0.66
Bone specific agent, n (%)	23 (57.5%)	13 (65.0%)	16 (80.0%)	0.48

*BMI* body mass index, *BMD* Bone Mineral Density, *S-Ca total* total serum calcium, *S-Ca ion* serum ionized calcium, *S-PTH* serum parathyroid hormone

### Fractures

**Fig. 2:**
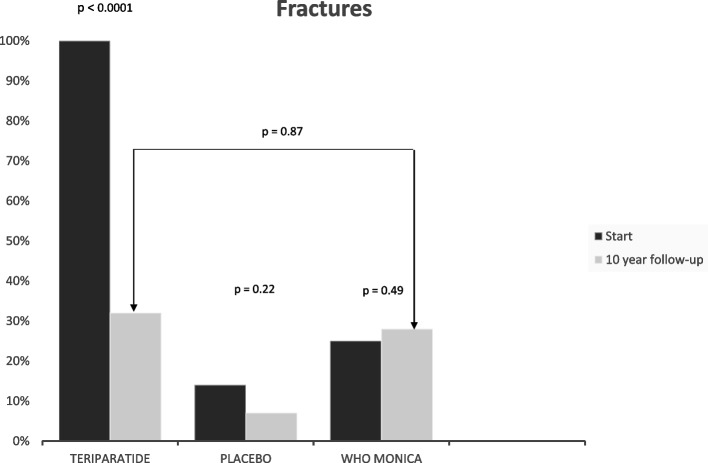
Percentage of women with fractures at treatment start and 10 years later for patients with osteoporosis treated with teriparatide, placebo and in women from the population sample, the WHO MONICA study, Gothenburg, Sweden. P-levels above the paired bars indicate within group comparison. The p-level with clambers indicates comparison between patients treated with teriparatide and the population sample, the WHO MONICA study, Gothenburg, at the 10-year follow-up

All women in the teriparatide study had suffered from at least one vertebral compression (100%) at start according to the inclusion criterion. Fractures declined during the follow-up of the teriparatide study group, from 100 to 35% (*p* < 0.0001), to rates comparable to the population sample (Fig. 2). Fractures in the WHO MONICA population sample were prevalent in 25% at start and 28% at the 10-year follow-up, not significant (Fig. 2).

Thirteen of the 20 women (65%) who completed the 10-year follow-up of the teriparatide treatment, did not experience any more fractures during follow-up. Seven of 20 (35%) patients suffered from one or more fractures, between 1.5 and 10 years after teriparatide start: four vertebral compressions, three forearm fractures, two upper arm, one hip and one rib fracture. Five patients had experienced a hip fracture before inclusion. At the follow-up, one of them was unable to participate, two had no new fracture, one of which was deceased after 8 years in the study. Of the remaining two patients, one had a rib fracture 9 years after treatment start and the other a new hip fracture 1 year and 8 months after treatment start, respectively. The latter died 2 years later. Of the 11 patients who died after treatment start, five had a hip fracture in addition to a rib and an upper arm fracture, respectively. One of them had the hip fracture 1.5 years after teriparatide treatment start and died 2 years after the fracture; two had a hip fracture 3 years after treatment start and died at 2 years and 3 years, respectively, after their fracture. The fourth and the fifth patient experienced a hip fracture six and 9 years after treatment start and died 2 years and 1 year after their hip fracture, respectively.

All subjects in the placebo group in the growth hormone study, underwent a spine X-ray at baseline, as well as after 18 and 36 months and the Genant score [19] was used for estimation of the vertebral height. No new vertebral compressions and no significant change in peripheral fractures were found in the placebo group during follow-up [20] (Fig. 2).

### Bone measure

**Fig. 3:**
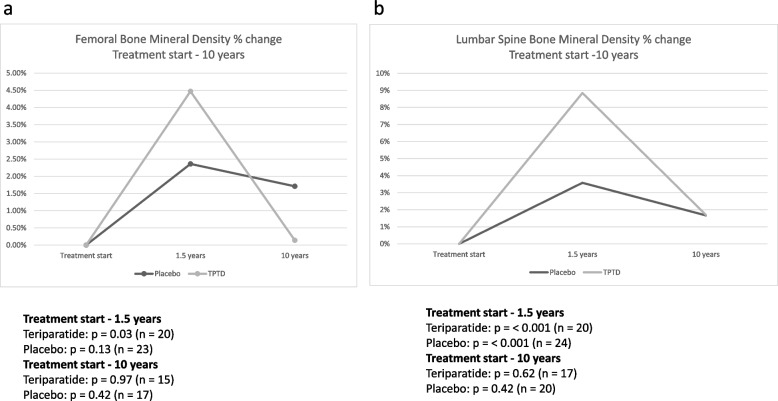
Percentage change in Femoral neck Bone Mineral Density (BMD) (**a)** and Lumbar spine BMD (**b**) from treatment start, to 1.5 and 10 years later with teriparatide and placebo treated patients with osteoporosis, respectively. P-levels within each treatment group and number of subjects at each occasion are shown below the diagrams. Mean treatment time was 18 months followed by other bone specific agents in comparable numbers during follow-up in the teriparatide and placebo group, respectively

The percentage change in BMD measured with DXA in the femoral neck and the lumbar spine at baseline, 1.5 and 10 years later, between teriparatide treatment and placebo-treated patients during the 10 years follow up, respectively, is shown in Fig. 3a and b. Teriparatide induced a significant increase after 1.5 years in both femoral neck (*p* = 0.03 versus start) and lumbar spine (*p* < 0.001 versus start) but BMD decreased thereafter to non-significant differences compared with treatment start. Lumbar spine BMD increased also in those who received placebo (*p* < 0.001 versus start) after 1.5 years, but not in femoral neck (*p* = 0.13 versus start), Fig. 3a and b. There was a comparable number of women who were treated with other bone specific agents after the 18 months of teriparatide or placebo, respectively.

### Quality of life

**Fig. 4:**
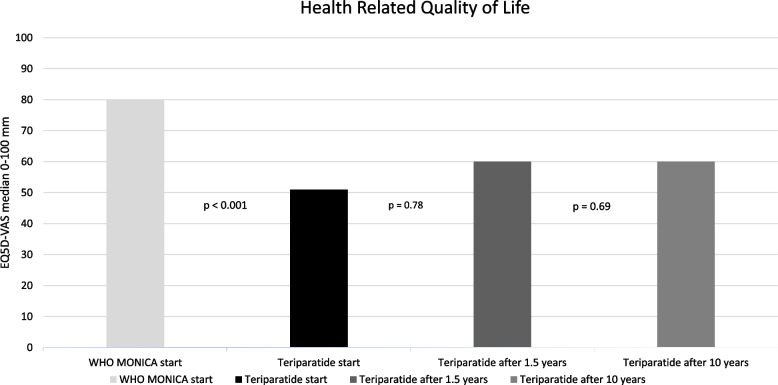
Health Related Quality of Life (HRQoL) for patients treated with teriparatide and for the population sample. EQ5D-VAS (median score between 0 and 100 mm). Comparison between the WHO MONICA population at start and teriparatide at treatment start (*p* < 0.001); between teriparatide treatment start and treatment cessation after 1.5 years (*p* = 0.78) and between teriparatide at start and after 10 years for the patients with osteoporosis, respectively (*p* = 0.69)

Total well-being, according to the EQ5D-VAS, was lower in patients with osteoporosis before treatment with teriparatide (median 51 mm), than in the WHO MONICA population sample (median 80 mm) (*p* < 0.0001), (Fig. 4). EQ5D-VAS did not improve after teriparatide treatment (*p* = 0.78) or at 10 years after teriparatide treatment start (*p* = 0.69).

**Fig. 5:**
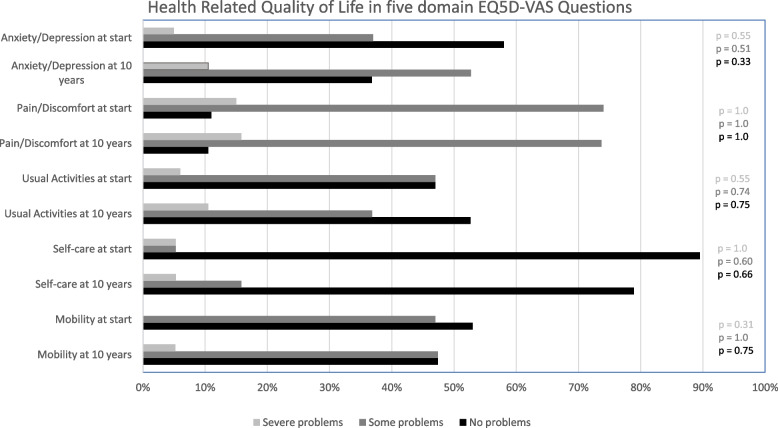
EQ5D-VAS scores of the women with osteoporosis treated with teriparatide at start and after 10 years (Severe problems, Some problems, No problems) in five different Health-Related Quality of Life (HRQoL) domains (Mobility, Self-care, Usual Activities, Pain/Discomfort, Anxiety/Depression). *P*-values for comparison between start and after 10 years are reported for each domain, respectively

In the five HRQoL domains most of the patients had some problems or no problems in daily life activities both at start of the teriparatide treatment and at follow-up, respectively. HRQoL, including pain/discomfort, was unchanged after 10 years (Fig. 5).

### Side effects of teriparatide

Five patients had side effects shortly after treatment start with teriparatide and did not complete the treatment. The side effects reported were pain in the limbs, dizziness and fatigue. One patient died before completing the treatment. The cause of death was a myocardial infarction 4 months after treatment start.

After the teriparatide treatment, bisphosphonates were given to 15 patients and denosumab was administrated to one patient depending on BMD or fracture history. In total 80% received bone specific agents after teriparatide. Four patients without any new fracture had low fall risk and a normal DXA were not prescribed bone specific treatment after the teriparatide treatment. The need for bone specific agent was reevaluated at each annual medical appointment. For comparison, there were 4.5% of the women in the WHO MONICA population sample who were treated with bone specific agents during the same time period of follow-up.

## Discussion

The main finding in this study was that the fracture rate decreased from 100 to 35% in all subjects who had been treated with teriparatide due to osteoporosis for a mean period of 18 months followed by other bone specific treatment during a 10-year follow-up. The fracture prevalence was, at follow-up, no different from women in the general population who were followed in parallel. Fracture prevalence increased in the population sample from 25 to 28% and treatment for osteoporosis was rare (4.5%). The results on fracture reduction after teriparatide treatment are in harmony with the original study [3], and the larger cohort EFOS and ExFOS studies [11, 13, 21]. These previous reports were, however, shorter in follow-up and without controls [11, 13].

A secondary finding was that HRQoL was not affected by treatment with teriparatide or other bone specific agents during the entire follow-up time. HRQoL in the teriparatide group was far lower (EQ5D-VAS median 51 mm at start and 60 mm later) than in the general population (median 80 mm) [18]. Previous results from the EFOS have shown an increased HRQoL after completed treatment with persistence of the higher HRQoL 36 months after treatment but without reaching the score norms from population studies in German women [22]. The present results differ from previous studies which might be due to a selection bias in the different reports. All patients in the present study had at least one vertebral compression as an inclusion criterion (100%) and it is likely the reason for their very low HRQoL compared with the general population. In spite of no new fractures after study start in 65% of these severely affected women, their HRQoL did not improve, neither during treatment, nor during follow-up. No improvements in the subscales for pain or in daily life activity (Fig. 5) were seen either. This indicates that vertebral fractures have a greater impact on HRQoL with a higher burden for a more prolonged period of time than previously thought or estimated.

Teriparatide treatment was effective in increasing BMD at the regions of interest after 1.5 years. This effect is related to the direct action on the bone by stimulating new bone formation, as was also seen in the present study [3, 23]. Teriparatide increased BMD in lumbar spine [3] and reduced the risk for new fragility fractures [13]. In an earlier large observational study of 4000 patients (DANCE study) a reduction was seen, in both vertebral and non-vertebral fractures [24]. A review study concluded that teriparatide has beneficial effects to BMD of the hip [25] which was also seen in the current study. However, the effect of the anabolic treatment with teriparatide diminished during the follow-up, irrespective of other bone specific agents up to 10 years later. BMD decreased after treatment cessation in both the teriparatide and the placebo group while the fracture frequency was markedly lower than before treatment with teriparatide. Hence, BMD could be considered an intermediate end point used as a tool for assessment of treatment effect or compliance during the active treatment period, but not as fully predictive of fracture incidence [26]. The essential outcome in the treatment of osteoporosis must be the hard end-point; fracture.

A report by Willers et al. underscores the under-treatment of subjects with a previous fracture [1]. The results from the population sample in present study also indicate under-treatment since the fracture frequency was 28% during the 10 years but only 4.5% had osteoporosis treatment. The benefit of treatment was clearly seen in the teriparatide treated patients as depicted in Fig. 2.

Osteoporosis is a disease that affects millions of patients every year. Fractures secondary to osteoporosis are costly for society, reduce the HRQoL, and raise mortality [27]. Despite national and international guidelines offering important help in the diagnosis and treatment of osteoporosis, fragility fractures are an increasing problem worldwide [1, 5, 28]. Monitoring, patients’ compliance and lifestyle changes are also important parts for a successful prevention of osteoporotic fractures and their serious consequences. Subjects who have suffered from a recent fragility fracture have higher risk to suffer a new fracture in the near future [5], and every osteoporotic fracture increases the risk of invalidity and death [27, 29]. Treatment with teriparatide resulted in an increase in BMD both in lumbar spine and femoral neck as well as a reduction of new fractures up to 10 years after treatment start.

According to the most recent guidelines for the treatment of postmenopausal osteoporosis, from the Endocrine Society, teriparatide and abaloparatide (another anabolic agent for osteoporosis) are recommended for patients with very high risk for fractures [28]. The American Association of Clinical Endocrinologists (AACE) recommends anabolic bone agents as first line treatment even for patients who cannot use peroral osteoporotic treatment such as bisphosphonates, which are considered more cost effective [5].

Patients included in this real-world study with long follow-up were seriously affected by their osteoporosis and fractures. The low results for EQ5D-VAS at start were unaltered at follow-up, after treatment with teriparatide. This speaks in favor of established osteoporosis being a substantially debilitating disease.

This is the only study, to our knowledge, of teriparatide treatment for osteoporosis with such a long follow-up time and in comparison with an age-matched population based control group from the same region followed in parallel. Furthermore, the placebo group from a randomized controlled, double-blind study of a similar patient group served as a control group for the patients with osteoporosis in the present teriparatide study. Except for those women who dropped out due to side effects of teriparatide, treatment compliance was high.

A limitation in this teriparatide study is the open-label treatment design and the lack of randomization. However, teriparatide was an approved treatment for severe osteoporosis with positive effects on the bone. The use of an additional placebo group, similar as the one from the growth hormone trial [4] used in this study, would not offer any new knowledge regarding BMD. Another limitation is the lack of X-ray verification of possible silent vertebral fractures in the teriparatide treated group. However, an X-ray was performed if the patient reported sudden back pain or if height was reduced by more than 2–3 cm since the previous annual visit. A decline in height is seen during the life span in most subjects in the population [16, 30]; therefore, it is unlikely that the observed mean 1 cm decline in height after 10 years could be an indicator for a missed vertebral compression fracture.

Teriparatide treatment seemed to be effective and tolerable. The formation of new bone, not only in the lumbar spine, but also in the femoral neck had to be preserved with antiresorptive treatment directly after finishing the anabolic treatment. Teriparatide is now available both as biosimilar and generic drug which makes it more accessible for de novo use.

## Conclusions

Anabolic hormonal treatment with teriparatide reduced fracture prevalence to similar levels as in the general population at 10-years follow-up. Teriparatide was effective and tolerable with improved bone mass in femoral neck and in lumbar spine but did not improve the HRQoL in these severely affected patients. In order to preserve HRQoL, fractures must be avoided in the first place. Teriparatide is recommended as a first line treatment in secondary prevention of osteoporosis.

## Data Availability

The datasets used and/or analyses during the current study are available from the corresponding author on reasonable request.

## Abbreviations

BMD: Bone Mineral Density
HRQoL: Health Related Quality of Life
WHO MONICA: World Health Organization MONItoring of CArdiovascular diseases
HRT: Hormone Replacement Treatment
DXA: Dual energy X-ray Absorptiometry
BMI: Body Mass Index
S-Ca total: Total serum calcium
S-Ca ion: Serum ionized calcium
S-PTH: Serum parathyroid hormone
S-Osteocalcin: Serum osteocalcin
EQ5D-VAS: EuroQol-5 Dimension Visual Analogue Scale
AACE: American Association of Clinical Endocrinologists
